# Anorgasmia following initiation of GLP-1 agonist

**DOI:** 10.1093/sexmed/qfaf047

**Published:** 2025-07-15

**Authors:** Vidya Visvabharathy, Sally MacPhedran, Katie Shupp, Benjamin King

**Affiliations:** Department of Obstetrics & Gynecology, Case Western Reserve University/MetroHealth Medical Center, Cleveland, OH 44109, United States; Department of Obstetrics & Gynecology, Case Western Reserve University/MetroHealth Medical Center, Cleveland, OH 44109, United States; Case Western Reserve University/MetroHealth Medical Center, Cleveland, OH 44109, United States; Case Western Reserve University/MetroHealth Medical Center, Cleveland, OH 44109, United States

**Keywords:** anorgasmia, GLP-1, semaglutide, liraglutide, obesity

## Abstract

**Introduction:**

According to the Obesity Medicine Association, obesity is a chronic, progressive, relapsing, multifactorial and treatable neurobehavioral disease. A small number of drugs have been approved by the Food & Drug Administration (FDA) for weight loss. Liraglutide, Tirzepatide, and Semaglutide are GLP-1 agonists and anti-diabetic drugs, and are some of the latest FDA-approved medications for weight loss. Up to 50% of patients experience gastrointestinal (GI) distress, a common side effect, while taking these medications. Other side effects include tachycardia, antibody-mediated angioedema, anaphylaxis, irritation at the injection site, and hypoglycemia. However, very little is known regarding sexual dysfunction as a side effect of GLP-1 agonists. The following case report discusses the potential negative impact on sexual function of these widely prescribed anti-diabetic, weight loss medications. The case report also discusses a proposed mechanism of action of GLP-1 agonists on sexual function.

**Aims:**

To report the sexual side effects and plausible mechanism of female anorgasmia from GLP-1 agonists.

**Methods:**

Chart review of the patient case was conducted. Consultation with a Doctor of Pharmacy was also requested for additional guidance on possible mechanisms of action of GLP-1 agonists on sexual function.

**Results:**

Several proposed mechanisms causing anorgasmia were theorized, the most likely being GLP-1 agonist vasoconstriction of smooth muscle, which results in reduced oxygen delivery and blood flow to the genitals. This may hinder genital engorgement and arousal, in addition to causing impairment of smooth muscle contractions essential for orgasms. Other mechanisms of GLP-1 agonist-induced anorgasmia may involve their signaling in the brain due to their presence in the hypothalamus. GLP-1 receptor modulation in the hypothalamus may decrease dopamine and norepinephrine signaling, which are neurotransmitters crucial for motivation, pleasure, and orgasm as well as disrupt pathways involved in sexual desire and arousal.

**Conclusion:**

Further investigation on the prevalence of sexual dysfunction with GLP-1 agonists and their mechanism of action is needed.

## Background

According to the Obesity Medicine Association, obesity is a chronic, progressive, relapsing and treatable multifactorial neurobehavioral disease. Obesity is defined as having a body mass index (BMI) of 30.0 or higher. The Centers for Disease Control (CDC) states that the prevalence of obesity among women in 2021-2023 was 41%^1^. These numbers are steadily increasing. Obesity has detrimental effects on multiple organ systems, including the genitourinary system which is essential to sexual function. The effect of obesity on sexual dysfunction is multifactorial.^2^ Obesity may affect an individual’s self-image, which negatively impacts sexual desire. Obesity may also physically impair an individual from performing sexual acts, causing shame, embarrassment, and discomfort. Additionally, comorbid conditions such as type 2 diabetes and hypertension, frequently associated with obesity, may cause both macro- and microangiopathic changes which reduce blood flow and sensation to the genitals.

A small number of drugs have been FDA-approved for weight loss. According to a Kaiser Family Foundation poll, 18% of women have used a weight loss medication in their lives.^3^ The latest among them, Semaglutide and Liraglutide, GLP-1 agonists and anti-diabetic drugs, received FDA approval for weight loss in 2021. These medications contribute to weight loss by direct activation of the hypothalamus and indirect activation of the vagus nerve, which reduces appetite and food intake.^4^ The medications may also implicate reward and motivation pathways influencing attitudes toward food. Prominent side effects of the medication have also been studied in depth. The most commonly reported side effects of GLP-1 agonists include gastrointestinal distress such as nausea, vomiting, diarrhea and constipation.^5^ Sexual dysfunction is not a known side effect of GLP-1 agonists.

The following case report demonstrates the potential negative impact on sexual function of widely prescribed anti-diabetic weight loss medications. We will describe a case where the onset of anorgasmia and hypoarousal in a postmenopausal female occurred after initiation of Liraglutide and Semagluatide therapy. The case report also discusses several proposed mechanisms of action of GLP-1 agonists on sexual function and recommendations for future investigation.

## Case report

A 71-year-old female with a medical history of hypertension, hyperlipidemia, osteoarthritis, sciatica, and depression presented to the sex medicine center for complaints of acute changes in arousal and orgasm. Her stated goal was to “wake up the clitoris”.

Approximately 5 months prior to presenting to the Sex Medicine Center, the patient was evaluated in a Weight Management clinic and was started on Liraglutide subcutaneous injections (1.8 mg daily). Within 2 weeks of initiating the medication, she noticed a change in her sexual function, specifically anorgasmia. After 2 months, the patient then switched to Semaglutide weekly injections due to Liraglutide-induced nausea. Her anorgasmia persisted while on Semaglutide.

A complete Bio-Psycho-Social-Relationship intake and sexual history was performed in the sexual medicine center. The sexual history noted issues with arousal and orgasm that began “suddenly” 5 months ago. Prior to this, she was having “the best sex of her life”, after discovering clitoral orgasms for the first time. She describes the change in her orgasm as though the “clitoris went to sleep all of a sudden” ⁓5 months ago. She did not feel sensation to the clitoris.

Her medical history consisted of several comorbidities that were overall non-contributory to the acute change. Her hypertension and hyperlipidemia were stable for >10 years and controlled with triamterene/hydrochlorothiazide 37.5-25 mg tablets and atorvastatin 20 mg tablets, respectively. She used 0.1 mg vaginal estradiol for genitourinary syndrome of menopause which she found to be helpful. She had been taking escitalopram 20 mg daily for several decades for depression and had not had any recent changes in her mood.

The patient was in a long term monogamous relationship with her husband, the same male partner (age 72) for 39 years. There was no history of infidelity or separation in her relationship. He had not had any change in his sexual function. The patient denied any external contributing factors to her issues, including children, work, parents, religion/culture, or finances.

### Work up

Physical exam revealed a white female, well appearing, in good spirits who was alert and oriented. On pelvic exam, a thin labia with loss of elasticity, a clitoral hood which retracted easily without adhesions, no vulvar tenderness with application of Q-tip were noted. Overall, the vagina was found to be well-estrogenized for a 71-year-old.

She underwent neurologic testing that demonstrated normal sensation to light touch, vibration, and temperature in the S2-4 region. Bulbocavernosus reflex and lower extremity deep tendon reflexes noted no deficits.

Her 10-year Atherosclerotic Cardiovascular Disease (ASCVD) risk of a cardiovascular event was found to be 17%, for which she was on a high-intensity statin, atorvastatin 20 mg daily. Blood work obtained included thyroid function tests, electrolyte panel, lipid panel, vitamin B12, and vitamin D. All labs returned normal. Prolactin was deferred.

### Treatment: education & medication

The patient was educated on common etiologies for anorgasmia, including hypoestrogenism, hypoperfusion related to peripheral vascular disease, decreased nerve sensitivity, and impaired pelvic floor muscle function. The treatment approach focused on improving blood flow and nerve sensation.

The patient was prescribed a topical cream composed of 6% L-arginine, 5% pentoxifylline, 0.05% erogloidmsylates, 1% sildenafil, 0.1% testosterone. She was instructed to apply the cream to her clitoris 10 min prior to sexual activity. An additional recommendation of self-stimulation with vibratory sensation 5-10 min, three times a week was given.

At the 3 month follow-up appointment, she noted no change in the orgasm response with the above treatments. On follow-up at the patient’s Weight Management clinic, Semaglutide was discontinued after 3 months secondary to constipation and Tirzepitide was started. After 2 weeks of being on Tirzepitide, the patient noted a return in her orgasms.

## Discussion

GLP-1 is an incretin hormone released after eating to stimulate the enteroendocrine L-cells and alpha cells of the pancreas to produce insulin in response to elevated blood glucose.^6^ This hormone affects glucose regulation in multiple ways. Release of insulin is accomplished via activation of the adenylate cyclase metabolic pathway, activating cyclic AMP (cAMP) and protein kinase A (PKA), which produces insulin from pancreatic beta cells. GLP-1 also decreases gluconeogenesis by inhibiting glucagon release from pancreatic alpha cells. Glucose sensitivity is also increased by downstream effects of cAMP and PKA, which inhibit ATP-dependent potassium channels and increase the activity of calcium channels. The influx of intracellular calcium ions activates glucose-induced membrane depolarization and cell sensitivity to glucose. Finally, GLP-1 stimulates epidermal growth factor (EGF), which triggers phosphatidylinositol-3 kinase (PI3K) to activate transcription factors that promote beta cell growth and inhibit beta cell apoptosis.^7^

GLP-1 agonists have pleiotropic effects on multiple organ systems throughout the body, causing lower blood pressure, blood glucose control, weight loss and maintenance. This is the first case report that posits an association between GLP-1 agonist therapy with sexual dysfunction. This association is supported by the temporal nature of the onset of symptoms with initiation of Liraglutide. The possible association is further supported by the resurgence of the patient’s orgasm after stopping use of GLP-1 agonist therapy and initiation of Tirzepatide. Of note, Tirzepatide is a dual-acting high-affinity glucose-dependent insulinotropic polypeptide (GIP) and low-affinity GLP1 agonist. We posit that Tirzepatide allowed for return of orgasms due to decreased activation of the GLP1 receptor as compared with high-affinity GLP1 agonists such as liraglutide and semaglutide.

We propose that there is a plausible pharmacological mechanism for the effects of GLP-1 agonists on sexual dysfunction.^8^ These medications have both peripheral and central effects on the central nervous system.

### Peripheral effects

GLP-1 receptor agonism has varying effects on peripheral tissue. GLP-1 agonists may affect sexual function and specifically orgasm via mediation of blood flow to smooth muscle and modulation of the autonomic nervous system.

GLP-1 acts on smooth muscle present throughout the body, including the genitals.^9^ Due to GLP-1 agonists’ effect on lowering systemic blood glucose, it is hypothesized that less glucose in systemic vasculature would lead to less vascular damage, less reactive oxygen species, and greater anti-inflammatory effects. As a result, these medications may actually have a favorable effect on genital blood flow, which would enhance orgasmic sensations. However, this patient had long-standing hypertension and likely peripheral vascular disease may have blocked any potential positive GLP-1 agonist response on genital blood flow.

### Central effects

The GLP-1 receptor is present in different areas of the brain, including the cerebral cortex, thalamus, hypothalamus, substantia nigra, and cerebellum. The hormone GLP-1 crosses the blood–brain barrier to influence appetite control and satiety. Due to the prolonged half-lives of GLP-1 agonists such as semaglutide and liraglutide, these medications may have more prolonged effects in the CNS.

GLP-1 receptors are found in areas of the brain involved in sexual function, such as the hypothalamus. The neurotransmitters dopamine and norepinephrine, crucial for motivation, pleasure, and orgasm are known to be produced/released in the hypothalamus during sexual excitation. GLP-1 agonist interaction in these areas may decrease dopamine and norepinephrine signaling by mediating the effects of leptin in cerebral areas implicated in appetite and fullness. This could result in a decreased perception of pleasure and orgasm. GLP-1 agonism may decrease activation in areas including the amygdala and frontal cortex, which are also implicated in sexual desire and arousal pathways. The exact effect of GLP-1 on the CNS is not yet identified. However, preclinical studies on the effects of GLP-1 and addiction show decreased rewarding effects of addictive substances with systemically injected GLP-1.^10^ In this patient, central effects of GLP-1 agonism may contribute to anorgasmia via decreased perception of pleasure, desire, and arousal.

Furthermore, there may be a sex hormone-mediated difference in GLP-1 sensitivity. The anorexic (appetite-suppressing) impact of GLP-1 receptors is attributed to their presence in the hypothalamus and brainstem. Estrogen also has anorexic activity, largely due to its presence in central regions. One animal study found that female rats portrayed a greater sensitivity to the food reward effects of central GLP-1 receptor activation, possibly due to sex hormone mediation. For this reason, GLP-1 activation in the reward center of females may provide a neuroanatomical basis for sex difference effects of GLP-1 agonism resulting in a decreased pleasure response.

## Conclusion

There are several potential areas of investigation with respect to sexual dysfunction and GLP-1 agonist therapy. One area for further research includes evaluation of the frequency of sexual dysfunction as a side effect of this therapy in men and women. Additionally, further investigation into the mechanism of sexual dysfunction via GLP-1 agonist therapy is needed. If the anorgasmia mechanism is related to decreased genital blood flow, the impact of PDE-5 inhibitors, which cause smooth muscle relaxation and vasodilation, may counteract this side effect. If the mechanism is posited to be via a central process that decreases dopaminergic transmission, then perhaps the impact of pro-dopaminergic agents such as flibanserin or bremelanotide may be considered. However, further research is needed to guide treatment options.
